# Feasibility of perfusion cardiovascular magnetic resonance in paediatric patients

**DOI:** 10.1186/1532-429X-11-51

**Published:** 2009-11-30

**Authors:** Emanuela R Valsangiacomo Buechel, Christian Balmer, Urs Bauersfeld, Christian J Kellenberger, Juerg Schwitter

**Affiliations:** 1 University Children's Hospital Zurich, Division of Paediatric Cardiology, 8032 Zurich, Switzerland; 2 University Children's Hospital Zurich, Division of Diagnostic Imaging, 8032 Zurich, Switzerland; 3 University Hospital Zurich, Clinic of Cardiology, Zurich, Switzerland

## Abstract

**Aims:**

As coronary artery disease may also occur during childhood in some specific conditions, we sought to assess the feasibility and accuracy of perfusion cardiovascular magnetic resonance (CMR) in paediatric patients.

**Methods and results:**

First-pass perfusion CMR studies were performed under pharmacological stress with adenosine and by using a hybrid echo-planar pulse sequence with slice-selective saturation recovery preparation. Fifty-six perfusion CMR examinations were performed in 47 patients. The median age was 12 years (1 month-18 years), and weight 42.8 kg (2.6-82 kg). General anaesthesia was required in 18 patients. Mean examination time was 67 ± 19 min. Diagnostic image quality was obtained in 54/56 examinations. In 23 cases the acquisition parameters were adapted to patient's size. Perfusion CMR was abnormal in 16 examinations. The perfusion defects affected the territory of the left anterior descending coronary artery in 11, of the right coronary artery in 3, and of the circumflex coronary artery in 2 cases. Compared to coronary angiography, perfusion CMR showed a sensitivity of 87% (CI 52-97%) and a specificity of 95% (CI 79-99%).

**Conclusion:**

In children, perfusion CMR is feasible and accurate. In very young children (less than 1 year old), diagnostic image quality may be limited.

## Introduction

Coronary artery disease (CAD) in paediatric patients may occur after surgery for congenital heart disease involving the coronary arteries, after surgery for congenital coronary anomalies or in patients with an inflammatory disease affecting the mid- and small-size arteries such as Kawasaki disease or Takayasu arteritis [1-3]. Therefore assessment of myocardial ischemia is important not only in the adult population, but increasingly in the paediatric population as well, in which early detection and therapy of CAD may help preventing irreversible myocardial dysfunction [4].

Exercise-electrocardiography, stress echocardiography, quantitative x-ray coronary angiography (QCA), single-photon emission computed tomography (SPECT) and positron emission tomography (PET) have been so far the available techniques to evaluate myocardial perfusion. QCA is still the reference standard for detecting or quantifying CAD. Radionuclide perfusion imaging suffers from some attenuation artefacts and suboptimal spatial resolution; both techniques necessitate exposure to ionising radiation. As children may need repeated evaluation during long-term follow-up, a non-invasive and radiation-free technique for evaluating the myocardial perfusion is advantageous. Perfusion cardiovascular magnetic resonance (perfusion CMR) using the first-pass kinetics of a gadolinium bolus for detecting myocardial ischemia has shown promising results in clinical trials in adult patients with known or suspected CAD [5-7]. In children perfusion CMR may present some technical challenges, due to higher heart rate (HR), shorter circulation time and high spatial resolution required. On the top of all these challenges patient's cooperation may be sometimes suboptimal, and younger children need to be examined in general anaesthesia.

Aims of this study were to assess the feasibility of performing perfusion CMR in paediatric patients and to determine the diagnostic performance of CMR compared to QCA.

## Methods

### Patient Population

Between January 2003 and June 2006, 47 consecutive patients underwent 56 CMR examinations for evaluation of myocardial perfusion. Seven patients had two examinations and one patient was evaluated three times. The median age was 12 years (range 1 month - 18 years) and the median weight 42.8 kg (range 2.6 kg - 82 kg). The cardiac diagnoses are listed in table 1. The category others included patients with Williams Beuren syndrome, Bland-White-Garland syndrome, Takayasu arteritis, chest pain, syncope, left ventricular dysfunction and arrhythmias. Perfusion CMR was clinically indicated for suspected CAD on the basis of patient's symptoms or for assessment after coronary arteries interventions as summarized in table 1. X-ray coronary angiographies had been either previously performed for similar clinical indications some time before perfusion CMR was available at our institution, or planned after perfusion CMR, if this exam was inconclusive (n = 2) or suspected a myocardial perfusion defect.

**Table 1:** Cardiac diagnoses/surgery and interventions on the coronary arteries

Patients	N = 56
**Cardiac diagnoses and procedures**	
Kawasaki disease	18
Arterial switch operation	12
Ross procedure	10
Cardiomyopathy/myocarditis	7
Others	9
	
**Interventions on the coronary arteries**	
None	24
Transfer of the coronary arteries during surgery for CHD	18
Direct coronary artery surgery	12
Coronary artery stenting	2

Coronary surgery included aortocoronary bypass to the left coronary artery in 5 patients, a venous patch augmentation of the left coronary artery in 4, an aortocoronary bypass to the right coronary artery (RCA) in 2 and reduction of a coronary aneurysm with patch augmentation of a nearby stenosis of the RCA in one. Two patients underwent catheter-guided stenting of a coronary stenosis following Kawasaki disease.

### Technique

#### Perfusion CMR

All CMR examinations were performed using a 1.5 T scanner (Signa MR/I EchoSpeed, General Electric Medical Systems, Milwaukee, Wisconsin;USA) and a phased-array cardiac coil. The images were acquired during breath holding in all patients, by asking the patients to stop breathing in end-inspiration (n = 38) or by stopping mechanical ventilation in the same position (n = 18). Patient's HR and ECG tracing were continuously monitored; blood pressure was measured at 2 minutes intervals.

The protocol included cine steady-state free precession sequences in a vertical and horizontal long-axis plane, as well as a stack of short-axis acquisitions covering the entire left ventricle for evaluation of left ventricular volumes and function. The steady-state free precession images were acquired at rest with following parameters: TE minimum, flip angle 45°, bandwidth 125 kHz, matrix 224 × 224, number of excitations 1, field of view 26-45 cm, slice thickness 6-8 mm with 1-3 mm gap depending on the body size, retrospective gating with 20 images reconstructed per cardiac cycle.

Myocardial perfusion was assessed, in accordance to our standard protocol, under pharmacological stress with intravenous adenosine in a dose of 0.14 mg/kg/min during 3 minutes. The myocardial signal increase during first-pass was evaluated during a GADOLINIUM bolus injection (Gadopentetate dimeglumine, Magnevist^®^, Schering, Berlin Germany) in a dose of 0.1 mmol/kg injected in a cubital vein through a large bore cannula at flow rate of 5 ml/sec and followed by a saline flush of 25 ml, flow 5 ml/sec. Five to seven slices were acquired every second heart beat in a short axis plane (slice thickness 8 mm, gap 4 mm) using an hybrid echo-planar pulse sequence with selective notch pulse preparation [5,6] with following parameters: repetition time 6.5 ms, echo time 1.5 ms, echo train length 4, flip angle 25°, bandwidth ± 125 kHz, matrix 128 × 128, rectangular field of view of 26 × 20 cm to 38 × 28 cm (depending on patient's size), number of excitations 1, acquisition every second RR interval. The images were acquired during a breath-hold of 60 cardiac beats (where patients were instructed to hold their breath as long as possible) or during suspended mechanical ventilation in children requiring general anaesthesia.

Late gadolinium enhancement imaging (LGE) was performed 10-15 minutes after injection (Gadopentetate dimeglumine, Magnevist^®^, Schering, Berlin Germany; total dose 0.2 mmol/kg) by acquiring images in long- and short-axis planes with a inversion-recovery-prepared gradient-recalled echo pulse sequence and with an adjusted inversion time (180-210 ms) in order to null viable myocardium. Acquisition parameters: echo time minimum full, matrix 256 × 192, number of excitations 2, flip angle 20°, bandwidth ± 31.25 KHz, 24 views per segment, rectangular field of view 26 × 20 cm to 38 × 28 cm depending on patient's size, slice thickness 8 mm, trigger delay depending on HR.

Image analysis was performed on a commercially available workstation (Advantage Windows 4.0, General Electric). The ventricular function was calculated as previously described with the MASS analysis software package (Magnetic Resonance Analytical Software System Version 4.0, MEDIS medical imaging systems, Leiden, Netherlands) [8]. First-pass and LGE images were reviewed as cine loops and stand-images respectively, by two experienced cardiac imagers (JS, EVB), blinded to the results of quantitative coronary angiography. Perfusion abnormalities were assessed qualitatively by visual comparison of the contrast enhancement in different myocardial regions and the final diagnosis was reached by consensus. A significant perfusion deficit was present if gadolinium wash-in was delayed in the subendocardial layer or transmurally, inducing a signal reduction in the myocardium that persisted throughout the entire first-pass of gadolinium, i.e. typically for 5-6 seconds. If a signal reduction was seen in the basal part of the interventricular septum only (membranous portion), this was not considered to represent a perfusion deficit. Small and transient signal reductions not extending over more than one slice were not considered as perfusion deficit.

#### X-ray coronary angiography

X-ray coronary angiographies were performed during cardiac catheterisation with a biplane Integris BH 5000 cardiovascular system (Philips Medical System, Leiden, The Netherlands). Selective coronary angiography was performed by hand injection of an ionized contrast agent (Iopamiro^®^, Sintetica SA, Mendrisio, Switzerland). In small children or in cases in whom anatomy precluded selective cannulation of the coronary arteries, contrast was injected into the aortic root at a dose of 1-1.5 ml/kg and with the maximal flow velocity allowed by the size of the catheter (1-2 ml/sec) by using a power injector. The images were acquired with a frame rate of 25/sec in the standard projections for visualisation of the right and the left coronary artery as previously described [9].

Quantitative coronary angiography (QCA). X-ray coronary angiographies were analysed using Inturis Cardio View software, Release 1.2 (Philips Medical Systems, Leiden, The Netherlands). Before measuring the diameter of the coronary arteries, calibration was obtained by comparing the diameter of the catheter shown on the angiographic images with the catheter diameter in French supplied by the manufacturer. In order to increase the accuracy of the measurements the external diameter of the catheter was measured when empty of contrast. Stenosis was defined as a narrowing of a coronary artery with a reduction of the vessel diameter of ≥ 50%.

An experienced cardiologist (CB) blinded to the results of perfusion CMR analyzed the QCAs.

### Statistics

Numerical data are reported as mean values and standard deviations or as median values and ranges where appropriate. Accuracy was evaluated by calculating sensitivity and specificity (and their 95% confidence interval) of perfusion CMR vs. QCA. A p value < 0.05 was considered as statistically significant. The Research Ethics Board of our institution approved the study. Parents gave their informed consent for the perfusion examination.

## Results

Fifty-six perfusion CMR examinations were performed in 47 consecutive patients. Perfusion CMR was successfully performed and diagnostic image quality was obtained in 54 of 56 examinations. The image quality of two examinations was insufficient, due to lack of compliance in a 10 years-old patient and to the very small body size (2.7 kg) in a 3 weeks-old newborn. The overall mean examination (scanning) time was 67 ± 19 min. Baseline mean heart rate (HR) was 78 ± 19 bpm, systolic blood pressure (BP) 102 ± 17 mmHg, diastolic BP 58 ± 13 mmHg. Peak HR under hyperaemia was 100 ± 19 bpm, systolic BP 99 ± 22 mmHg, diastolic BP 53 ± 15 mmHg.

Perfusion CMR was performed in general anaesthesia in 18 patients. In 23 examinations the acquisition parameters were modified according to patient's size. Slice thickness was reduced from 8 to 7 mm in 16, the gap between slices from 4 to 3 mm in 19 studies and to 2 mm in 4. A field of view smaller than 30 × 23 cm was used in 19 studies, all patients younger than 10 years. The injection velocity of the CM bolus was adapted to the size of the cannula and reduced from 5 to 4 ml/sec in 4 cases, to 3 ml/sec in 5 and to 2 ml/sec in the youngest child.

LGE was performed in addition to the first-pass acquisitions in 13 cases. Indication for performing LGE acquisitions consisted of a patient's clinical history suggesting potential occurrence of myocardial scar, i.e. a positive history for myocardial infarction, myocarditis or cardiomyopathy. In one case the image quality of LGE was insufficient due to arrhythmias.

Minor technical problems occurred during 3 examinations and consisted of triggering difficulties, software problems, and initial IV line dysfunction in one patient each. The technical difficulties were transient and the examination could be successfully concluded in all 3 cases.

All examinations were performed without any significant medical complications. In one child under general anaesthesia atropine was given to treat bradycardia (not related to adenosine), one patient showed a self-limiting mild arterial hypotension, and a girl with a total occlusion of left anterior descending coronary artery (LAD) experienced chest pain during adenosine infusion, but remained haemodynamically stable.

### Left ventricular volumes and function

Data about left ventricular (LV) volume and function were available for 53 examinations. Mean LV end-diastolic volume (LVEDV) was 79.9 ± 24.6 ml/m^2 ^and mean ejection fraction (EF) was 54.5 ± 9.8%. A dilated LV (LVEDV > 100 ml/m^2^) [8] was found in 4 patients: two of them had a combined aortic valve disease, one a LV subvalvular outflow tract obstruction, and another one was performing high-endurance sports.

Reduced function with EF < 45% was measured in 9 patients: 7 of these presented with abnormal coronary arteries, including Kawasaki disease in 5, Bland-White Garland syndrome in one, and LAD narrowing after arterial switch operation in one. Five of these patients had previous surgery of the coronary arteries and 3 had a history of myocardial infarction.

### Myocardial perfusion

**Figure 1:**
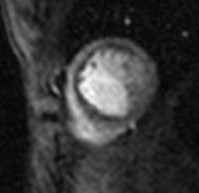
**Subendocardial perfusion defect in the territory of the left coronary artery in a 15 years-old girl with an occluded intracoronary stent**.

A normal perfusion CMR study was observed in 38 exams. Perfusion was abnormal in 16 examinations with a perfusion deficit in the territory of LAD in 11, of RCA in 3, and of left circumflex coronary artery (LCX) in 2 cases (Figure 1). Assessment of viability with LGE was abnormal in 6 examinations. Thus, by combining perfusion and viability information, 6 abnormal perfusion exams were caused by the presence of myocardial scars: in 4 cases with known myocardial infarction (2 Bland-White-Garland syndrome, 1 Kawasaki disease with LAD stenosis, and 1 postoperative infarction after coronary artery surgery in arterial switch operation for transposition of the great arteries), in one patient with hypertrophic cardiomyopathy, and in one patient with a history of myocarditis. In a patient with hypertrophic cardiomyopathy a diffuse subendocardial defect was detected during the hyperaemic first pass study and was interpreted as relative ischemia due to severe myocardial hypertrophy.

### Quantitative X-ray coronary angiography

QCA was performed in 31 patients. The median time interval between perfusion CMR and QCA was 13.5 ± 19 months. Nineteen angiographies were performed within a time interval of less than 3 months (median 2.4 months). Normal coronary arteries were found in 20 patients and a coronary artery stenosis in 8, consisting of a LAD stenosis in 5, RCA stenosis in 2, and stenosis of the LCX in one case. Aneurysmatic enlargement of the coronary arteries was described in 3 children after Kawasaki disease.

### Accuracy

**Figure 2:**
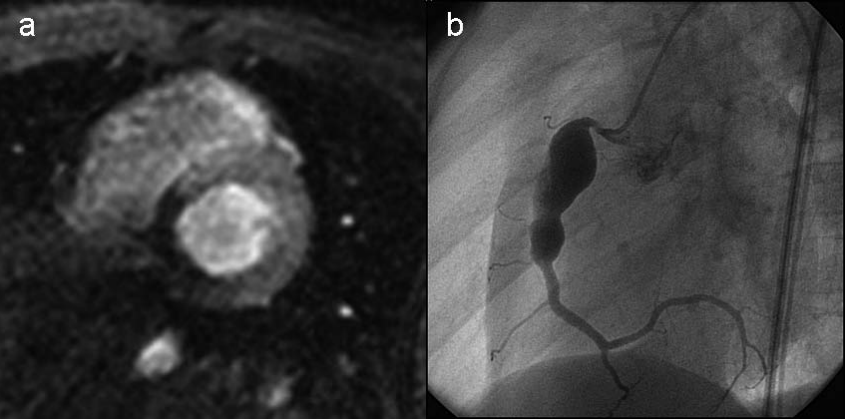
**2a. Transmural perfusion defect in the territory of the right coronary artery in a 7 years-old boy after Kawasaki disease**. 2b. Coronary angiography showed a giant aneursym of the right coronary artery, but failed to demonstrate a stenosis at the distal end of the aneurysm, which was subsequently depicted intraoperatively, at time of surgical resection of the aneurysm.

Perfusion CMR findings were concordant with QCA in 29 and discordant in 2 cases (Table 2). In one patient after Kawasaki disease perfusion CMR showed abnormal perfusion in the inferior wall segments, but QCA described a giant aneurysm of the RCA without stenosis (case * in table 2). Intraoperatively a significant stenosis at the distal end of the aneurysm was identified and a patch augmentation of the RCA performed (Figure 2). In one patient with a normal perfusion CMR a 60% narrowing of the origin of the RCA was described at QCA (case ** in table 2); intraoperatively the coronary arteries were found to be normal.

**Table 2:** Correlation of the findings between perfusion-CMR and coronary angiography

		**Quantitative coronary x-ray angiography**			
	
**Perfusion-CMR**		**LAD**	**LCX**	**RCA**	**Normal**
	
	LAD	4			
	
	LCX		1		
	
	RCA			2	1*
	
	Normal			1**	23

LAD: left anterior descending artery, LCX: left circumflex artery, RCA: right coronary artery

* Stenosis at the distal end of a giant aneurysm, not seen at coronary angiography but demonstrated intraoperatively

** Coronary angiography depicted a 60% narrowing of the right coronary artery, no significant stenosis was found at surgery

Compared to QCA perfusion CMR showed a sensitivity of 87% (CI 52%-97%), and a specificity of 95% (CI 79%-99%). Agreement between the perfusion CMR results and the intraoperative findings was 100%.

## Discussion

The clinical impact of perfusion CMR as a non-invasive technique for the assessment of ischemic heart disease is rapidly increasing [10]. In children CAD is much less frequent than in adults and mainly related to congenital heart defects and their surgical repair, or to inflammatory disease affecting the coronary arteries. Nevertheless, particularly young patients may benefit from the advantages of perfusion CMR, such as the lack of exposure to ionising radiation [11], and its non-invasiveness overcoming the potential complications of cardiac catheterisation [12].

To our knowledge, this is the largest study reporting the use of perfusion CMR in paediatric patients. Our data demonstrate a feasibility of 96%, and a high accuracy, with a sensitivity of 87% and a specificity of 95%. These results are similar to those reported in adults in single-centre [5,7] and multi-centre trials [6,13]. The largest multicentre multivendor perfusion CMR study published recently has shown that perfusion CMR has a better diagnostic performance than SPECT, when results from QCA were used as the reference standard [14]. In the paediatric population a comparison with nuclear perfusion techniques is problematic. PET and SPECT are not well-established diagnostic tools, since ethical reasons preclude the establishment of normal database in infants and children [13]. Moreover, selected congenital conditions such as cyanosis, with possible hypoxia-related effects on the myocardium may preclude a correct interpretation of the perfusion metabolism pattern shown by radionuclide techniques [15].

The paediatric experience reported so far in assessing ischemic disease with perfusion CMR is scarce [16,17]. One study demonstrated the feasibility of performing CMR coronary angiography and late enhancement imaging in children [17]. Prakash et al. reported their preliminary experience showing good agreement between perfusion CMR and QCA or SPECT in a limited number of patients and without providing data on sensitivity and specificity. The authors concluded that further validation of the technique with additional studies is needed [16].

### Technical considerations

Several larger perfusion CMR trials established key components of perfusion pulse sequences [5,14]. However, paediatric patients require a particular technical approach with some modifications of pulse sequences parameters. Children have the characteristics of a small body size, higher HR, and, at least in younger subjects, limited compliance. In younger patients examined in general anaesthesia, mechanical ventilation with the option of suspend ventilation during image acquisition is crucial for obtaining a sufficient study quality. Correspondingly, we studied all patients younger than 8 years in general anaesthesia and during breath-holding. This recommendation seems to be valid not only for perfusion CMR, but also for CMR coronary angiography and LGE imaging in children [17].

We utilized a gadolinium dose of 0.1 ml/kg, which has been demonstrated to give the best results in adults, also in all our paediatric patients. In contrast, the gadolinium injection rate needed to be reduced and adapted to the size of the intravenous cannula in 10 patients. Since Gebker et al. showed that myocardial enhancement and upslope are largely independent from the injection rate of gadolinium bolus as long as the injection speed is not below 3 ml/sec [18], we targeted to keep injection speed above this limit. Correspondingly in the only case, in which we had to use a slower injection rate of 2 ml/sec, the images resulted not diagnostic.

**Figure 3:**
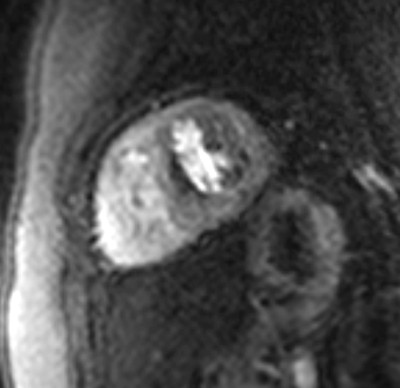
**Susceptibility artefacts ("dark rim") presenting as subendocardial hypointense region in the basal septum in a 5 years-old boy**.

Susceptibility artefacts originating at the interface between blood (high gadolinium concentration) and the myocardium (low gadolinium concentration) may occur more often in small hearts with thin walls, in which fewer pixels are imaged transmurally. Kellman et al. demonstrated larger artefacts at lower spatial resolution [19]. It is therefore crucial to reduce the field-of-view and thus, increase spatial resolution in paediatric studies (even allowing for wrap-around artefacts close to the LV). In the current study we observed a thin subendocardial hypointense region in the septal segments of 4 patients during hyperaemic first-pass of gadolinium (figure 3). Based on the experiences from this study, these signal deficits in the septum, particularly when restricted to the basal portion of the septum, should not be diagnosed as ischemia.

High HR in children, caused by both, an elevated baseline HR and a more sensitive response to adenosine, which we could consistently observe in our study, require an efficient and robust ECG-triggering system. The relatively new vector cardiac gating systems usually yield to a good triggering, even in higher heart rate, without need for any additional adjustment.

In spite of all these challenges, by carefully tailoring the examination conditions in each individual case, we were able to obtain a diagnostic imaging quality in 96% of the examinations. Perfusion CMR was misleading in two cases: in 3 weeks-old newborn weighting 2.7 kg in whom the rate of gadolinium injection needed to be reduced to 2 ml/sec, and in another child unable to be compliant to hold his breath.

**Figure 4:**
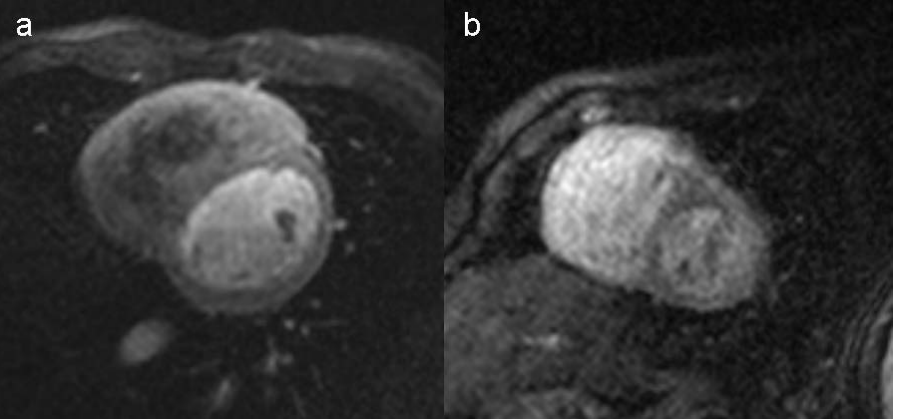
**Comparison of image appearance between a 17 years-old (a) and in a 3 years-old (b) patient**. 4a. Imaging parameters: matrix 128 × 128, FOV 35 × 26 cm, slice thickness 8 mm, gap 4 mm. Injection rate of gadolinium 5 ml/sec. Heart rate at image acquisition 90/min. 4b. Imaging parameters: matrix 128 × 128, FOV 25 × 19 cm, slice thickness 7 mm, gap 3 mm. Injection rate of gadolinium 3 ml/sec. Heart rate at image acquisition 87/min.

On the base of our initial experience described in this paper, we are now able to provide some recommendations on how to optimise the preparation of the patient and the acquisition of the images. Firstly, we recommend performing perfusion CMR only in children older than 1 year or weighting more than 10 kg. The possibly largest i.v. cannula should be inserted in the forearm of the patient, allowing gadolinium injection rate of at least 3 ml/sec. Secondly, image acquisition should be performed during breath-holding in conscious and anaesthetized patients. Thirdly, the same gadolinium dose of 0.1 mmol/kg as in adult can be used. Fourthly, spatial resolution can be optimise by reducing slice thickness and field of view below 30 × 23 cm, if a matrix of 128 × 128 is used, keeping in mind that an ideal in plane resolution in children might be 1.5-2.5 × 1.5-2.5 mm (figure 4). Finally the robustness of the ECG gating at higher heart rate can be tested by running the sequence a rest and without gadolinium injection, meanwhile the patient can exercise with breath-holding. Image acquisition every second heart beat provides enough preparation time for the preparation pulse and no further modifications are needed for heart rate.

### Limitations of the study

This is a retrospective study concealing some limitations. Firstly, our data present a rather wide range of time intervals between perfusion CMR and QCA. The reason for this is that some QCA data used for comparison had been obtained before perfusion CMR was available as a diagnostic technique in our institution. In these patients catheterisation was not repeated if no clinical suspicion of ischemic heart disease was present at perfusion CMR. Other patients were selected for QCA only, when perfusion CMR findings were abnormal. This more clinically oriented approach may have introduced a selection bias in the patient group undergoing QCA, and may have influenced accuracy. This kind of potentially serious limitation is often encountered in paediatric research, as in children ethical reasons preclude the liberal use of invasive examinations or anaesthesia for research purposes.

Secondly, as this study was performed in children, we sought to keep the examinations as short as possible and the patients compliant. Therefore, LGE was not performed consistently in every case, but only if indicated for clinical reasons or if perfusion CMR findings suggested the presence of ischemia and/or scar. Nevertheless, in spite of having applied this single perfusion CMR pulse sequence for evaluation of ischemia, we found a high sensitivity and specificity for the detection of CAD. However, in clinical practice with the suspicion of myocarditis or a storage disease, LGE should be performed as the preferred CMR approach [20].

Finally, as the CMR scans were performed for clinical reasons, we endeavoured to keep the sequence robust and performed only careful changes in the acquisition parameter for adapting them to the patient's size. Testing different "paediatric protocols" in a standardized way was not the aim of our study, but may be needed in the future for further optimisation and validation of the method in the paediatric population

## Conclusion

In the paediatric population, perfusion CMR is feasible and accurate. Age and body size are still limiting factors, and very young children, e.g. < 1 year, are considered suboptimal candidates for this non-invasive technique. However, in older children with suspected coronary alterations perfusion CMR can be recommended as a highly valuable first test as well as an important adjunct to invasive x-ray coronary angiography.

## Competing interests

The authors declare that they have no competing interests.

## Authors' contributions

EVB conceived and designed the study and gave a major contribution to the draft of the manuscript and its revision. CB performed and analysed the data of coronary angiography and contributed to the draft of the manuscript. UB helped to design the study and to revise the manuscript. CJK was involved in data acquisition and draft of the manuscript. JS conceived and designed the study and gave a major contribution to the draft of the manuscript and its revision. All authors read and approved the final manuscript.
